# *In vitro* cell culture model of human nasal-associated lymphoid tissue (NALT) to evaluate the humoral immune response to SARS-CoV-2 spike proteins

**DOI:** 10.1016/j.sjbs.2021.04.051

**Published:** 2021-04-24

**Authors:** Waleed H. Mahallawi, Talal M. Aljeraisi

**Affiliations:** a Department of Medical Laboratory Technology, College of Applied Medical Sciences, Taibah University, Madinah 41541, Saudi Arabia^1^; b Otorhinolaryngology, Head& Neck Surgery Department, Faculty of Medicine, Taibah University, Madinah, Saudi Arabia

## Abstract

To date, coronavirus disease 2019 (COVID-19) continues to be considered a pandemic worldwide, with a mild to severe disease presentation that is sometimes associated with serious complications that are concerning to global health authorities. Scientists are working hard to understand the pathogenicity of this novel virus, and a great deal of attention and effort has been focused on identifying therapeutics and vaccines to control this pandemic.

**Methods:**

This study used tonsils removed from twelve patients who underwent an elective tonsillectomy in the ear, nose, and throat (ENT) department at Saudi Germany Hospital, Madinah, Saudi Arabia. Tonsillar mononuclear cells (MNCs) were separated and co-cultured in RPMI complete medium in the presence and absence of viral spike (S) proteins (the full-length S, S1 subunit, and S2 subunit proteins). Enzyme-linked immunosorbent assay (ELISA) was used to measure secreted antibody concentrations following stimulation.

**Results:**

The *in vitro* human nasal-associated lymphoid tissue (NALT) cell culture model was successfully used to evaluate the humoral immune response against SARS-CoV-2- S protein. Significant (p < 0.0001, n = 12) levels of specific, anti-S IgG, IgM, and IgA antibody responses were detected in cells culture supernatanat folloeing stimulation with the full-length S protein compared with unstimulated cells. In contrast, S1 and S2 subunit proteins alone failed to induce a mucosal humoral immune response following tonsillar MNC stimulation.

**Conclusion:**

We demonstrated a successful human NALT *in vitro* cell culture model that was used to study the mucosal humoral immune response to the SARS-CoV-2 S protein. This model could be advantageous for the in-depth study of cellular immune responses to the S protein and other viral antigens, such as nucleocapsid and matrix antigen. The S protein appears to be the important viral protein that may be able to mimic the natural infection process intranasally and should be studied as a component of a candidate vaccine.

## 1 Introduction

The outbreak of the novel coronavirus disease 2019 (COVID-19), which is caused by severe acute respiratory syndrome coronavirus 2 (SARS-CoV-2), has rapidly spread and is currently considered a global pandemic (Eckerle and Meyer, 2020). Numerous institutions and public research organizations have focused their efforts on identifying effective therapeutics to treat COVID-19 (A et al., 2020). Presently, various approaches for producing a successful immune response against SARS-CoV-2 in humans are being explored by many scientific communities, with massive levels of support from both government and private sectors.

One method for designing a vaccine is the administration of one or more SARS-CoV-2 subunit antigens, as either purified proteins or in the form of viral, RNA, or DNA vaccine vectors capable of producing these proteins. Potential targets for immunization include the essential proteins that bedeck the SARS-CoV-2 surface, including the spike (S), envelope (E), matrix (M), and nucleocapsid (N) proteins (Salvatori et al., 2020).

Mucosal immunity represents an important component of the immune system (Kiyono and Fukuyama, 2004). Among the various mucosal administration sites, the nasal cavity represents one of the most striking compartments. The nasal cavity comprises a vastly vascularized epithelial layer with a huge surface area that can be utilized for vaccine delivery (Kang et al., 2013). Nasal-associated lymphoid tissue (NALT, a component of mucosa-associated lymphoid tissue that is embedded in the nasal submucosa) is considered to be the central inductive location for immune responses to both natural infections and vaccinations that utilize the nasal route. NALT is known to be an important immune compartment for both mucosal and systemic immunity against upper respiratory tract (URT) pathogens (Wu and Russell, 1997, Kiyono and Fukuyama, 2004, Beniova et al., 2014). NALT has been shown to play a significant role in immune protection against influenza infection (Tamura and Kurata, 2004). Tonsils are components of the mucosal immune system, consisting principally of B-cells, which constitute approximately 65% of tonsillar tissue, in addition to nearly 30% CD3^+^ T-cells and approximately 5% macrophages. Tonsillar T-cells primarily belong to the CD4^+^ subset, which comprises greater than 80% of all CD3^+^ T-cells (Boyaka et al., 2000, Sada-Ovalle et al., 2012).

Mucosal vaccination represents an encouraging alternative to parenteral vaccination routes. Mucosal vaccination is non-invasive and able to provoke robust local and systemic immune responses in mucosa-associated lymphoid tissue. Recent research has focused on improving mucosal vaccines, such as intranasal vaccines targeting respiratory tract infections (Lycke, 2012). Furthermore, they are the primary stimulation location for immunity induced by intranasal vaccines, such as live attenuated influenza vaccines (LAIV). Because immune tolerance represents a primary role of the mucosal immune system, mucosal vaccinations are typically designed using either a live attenuated vaccine or an inactivated virus or subunit vaccine antigen combined with an adjuvant (Belshe et al., 2007).

Viral S proteins function by binding host receptors and have been viewed as promising targets for both vaccine and antiviral therapeutic development. Previous studies examining SARS-CoV and Middle East respiratory syndrome coronavirus (MERS-CoV) have shown that vaccines based on the viral S protein can produce antibodies that block virus binding and fusion to neutralize viral infections (Du et al., 2006). The ongoing spread of SARS-CoV-2 represents a worldwide health disaster. The burden on governments continues to escalate with the increasing number of new cases due to the rapid infectivity of the virus. Numerous cases of reinfection have been described, although protection from reinfection has also been described (Mahallawi and Al-Zalabani, 2021). Therefore, the development of a vaccine against this novel virus may be a major preventive measure that can protect people against infection.

In this study, we developed a novel, human, NALT-derived cell culture model of tonsil tissue as an alternative to currently used cell lines to study the humoral immune response against the newly emerged SARS CoV-2 viral proteins. This model mimics the natural site of SARS-CoV-2 infection. We used the NALT cell culture model to observe the immunogenicity of various SARS CoV-2 proteins. To develop this cell culture model, we isolated a single-cell suspension from fresh NALT derived from tonsillar tissues and measured the tendency of NALT-derived lymphocytes to produce specific antibodies against viral proteins that were used to stimulate the cells. Enzyme-linked immunosorbent (ELISA) was used to detect the induced antibody in cell culture supernatants.

## 2 Materials and methods

The study was approved by the Taibah University Ethical Committee (IRB No. MLT 2020030).

### 2.1 Tonsillar samples

A total of twelve patients who underwent elective tonsillectomy at the ear, nose, and throat (ENT) department at Saudi Germany Hospital, Madinah, Saudi Arabia (from mid of October to mid of December 2020), were chosen for this study they were 7 males and 5 females. Signed informed consent was obtained from all adult patients and the parents of children patients (age range: 2–23 years, mean age: 9.7 years).

### 2.2 Inclusion criteria

Patients who suffered only from snoring or obstructive sleep apnea were included in this study.

### 2.3 Exclusion criteria

Patients with recurrent tonsillitis were excluded. Patients with any history of previous SARS-CoV-2 infection were also excluded.

After the surgery, the tonsils were immediately placed in HANKS transport medium containing glutamine (2 mM), penicillin (50 U/mL), and streptomycin (50 μg/mL) (Sigma-Aldrich) and transferred to the laboratory for the processing and isolation of tonsillar mononuclear cells (MNCs).

### 2.4 Isolation of tonsillar mononuclear cells

Cell suspensions were prepared using a modified cell suspension protocol based on a previously described method (Mahallawi et al., 2013). In brief, under sterile conditions, tonsillar samples were treated within one hour of surgery. The tissues were cut into small portions, moved to an 8-cm-diameter sterile Petri dish, and checked grossly. Each sample was disrupted and shredded by a particular sterilized blade. The sample tissues were then lifted to release the cells into RPMI complete medium (RPMI 1640 medium with HEPES supplemented with 10% fetal bovine serum, 2 mM glutamine, 50 U/mL penicillin, and 50 μg/mL streptomycin; Sigma Aldrich). The cell suspension was passed through a 70-μm sterile nylon mesh to remove any tissue debris. MNCs were isolated using Ficoll-Paque (Premium GE Healthcare, United Kingdom) gradient centrifugation (400 × *g* for 30 min). The cells were washed two times in sterile phosphate-buffered saline (PBS), then resuspended in 5 mL RPMI complete medium to culture the cells. Each cell suspension was adjusted to a concentration of 4 × 10^6^ cells/mL.

## 3 Recombinant SARS-CoV-2 S proteins

### 3.1 SARS-CoV-2 spike protein (S1 + S2)

A DNA sequence encoding the full-length SARS-CoV-2 S protein (S1 + S2 ECD; Val16–Pro1213) with a polyhistidine tag at the C-terminus (S1 + S2-ECD-His, Sino Biological, Beijing, China) was expressed and the resultant protein was reconstituted in sterile phosphate-buffered saline (PBS). The S protein was expressed in HEK293 cell and consisted of 1209 amino acids.

### 3.2 SARS-CoV-2 spike S1-His recombinant protein

A DNA sequence encoding the SARS-CoV-2 S1 subunit (S1 ECD, Val16–Arg685) was expressed in HEK293 cells with a polyhistidine tag at the C-terminus and consisted of 681 amino acids. (Sino Biological, Beijing, China)

### 3.3 SARS-CoV-2 spike S2 ECD-His recombinant protein

A DNA sequence encoding the SARS-CoV-2 S2 subunit (S2 ECD, Ser686–Pro1213) was expressed in Baculovirus insect cells with a polyhistidine tag at the C-terminus and consisted of 539 amino acids. (Sino Biological, Beijing, China)

### 3.4 Cell counting and culture

A 10 µL MNC suspension was transferred to a hemocytometer, and the cells were counted using a light microscope at 40 × magnification. All cells in the 1 mm^2^ center square of the grid were counted.

## 4 Cell culture and NALT MNCs

### 4.1 Stimulation for antibody production

Following the separation of NALT MNCs, each cell suspension was adjusted to contain 4 × 10^6^ cells/mL. NALT MNCs were co-cultured in RPMI complete medium in the presence and absence of S proteins (full-length S, S1 subunit, or S2 subunit proteins). Various concentrations of S proteins were used as antigens to stimulate lymphocytes derived from NALT tissues. Unstimulated cells were used as the negative control. A 250 µL volume of each MNC suspension was cultured in a sterile 96-well cell culture plate (Costar; Corning, Corning, NY, USA) and placed in a humidified incubator in a 5% CO_2_ atmosphere at 37 °C. Cell culture supernatants were collected at various time points and stored at − 70 °C until analysis. ELISA was used to measure the antibodies induced in response to S protein stimulation.

### 4.2 Enzyme-linked immunosorbent assay (ELISA)

ELISA used in the current study performed according to the previously described method (Mahallawi, 2021). In brief, a 96-well ELISA plate (Costar; Corning, Corning, NY, USA) was coated with 2 µg/mL of SARS-CoV-2 recombinant S protein (Sino Biological, Beijing, China). The SARS-CoV-2 S protein was reconstituted in PBS, pH 7.2, and the plates were coated with 100 µL/well of SARS-CoV-2 S protein. The plates were then covered with an adhesive seal and incubated overnight at 4 °C. The plates were washed five times with washing buffer (PBS containing 0.05% Tween-20; Sigma-Aldrich, St. Louis, MO, USA), using an automated microplate washer (Elx50; Bio Tek, Winooski, VT, USA). The plates were then blocked with 150 µL/well blocking buffer (PBS containing 0.05% fetal bovine serum that was heat-inactivated at 56 °C for 60 min; Sigma-Aldrich) for one hour at room temperature. Cell culture supernatant samples were then diluted (1:5) with blocking buffer, and 100 µL was added to each well. The plates were incubated again for 30 min at room temperature and washed five more times with washing buffer. Specific alkaline phosphatase-conjugated goat anti-human IgG, IgM, and IgA secondary antibodies at 1:1,000, 1:2,000, and 1:1,000, respectively, diluted in blocking buffer (Sigma-Aldrich), were added at 100 µL/well, and the plates were incubated at room temperature for 30 min before being washed five times with washing buffer. Finally, 100 µL/well of the ready-prepared substrate, p-nitrophenyl phosphate (p-NPP, Sigma-Aldrich), was added. The plates were maintained in the dark, away from direct light, until color developed. After 30 min, 100 µL of stopping solution (1.2 N sodium hydroxide, Reagecon, UK), was added to all wells to terminate the reaction. The optical density at 405 nm (OD_405_) was measured using a microplate reader (ELX800; BioTek).

The sample was defined as ELISA-antibody-positive if the OD_405_ value was three standard deviations (SDs) above the mean value of the negative control.

### 4.3 Statistical analysis

All calculations and statistical analyses were performed using GraphPad Prism statistical software (version 9, USA). Data are expressed as the mean ± standard error of the mean (SEM). Comparisons between two groups were performed using a paired *t-*test, and p < 0.05 was considered significant.

## 5 Results

### 5.1 Determination of the optimal SARS-CoV-2 S protein concentration necessary to stimulate NALT MNCs

SARS-CoV-2 S proteins were used at varying concentrations to determine the optimal concentration necessary to stimulate NALT MNCs. We tested a range from 5 to 40 µg/mL. We used a hemocytometer to count the lymphocytes before and after stimulation to determine cell viability. A protein concentration of 20 µg/mL S (full-length) protein was determined to be the optimal concentration for MNC stimulation. Lower protein concentrations failed to stimulate tonsillar MNCs. When we increased the protein concentration above 20 µg/mL, cell viability decreased, indicating that higher concentrations were toxic and led to cell death. Unfortunately, the S1 and S2 subunits failed to stimulate the induction of a humoral response in tonsillar MNCs at all tested concentrations (data not shown).

### 5.2 SARS-CoV-2 S (full-length) protein induced a strong humoral immune response in NALT MNCs

**Fig. 1:**
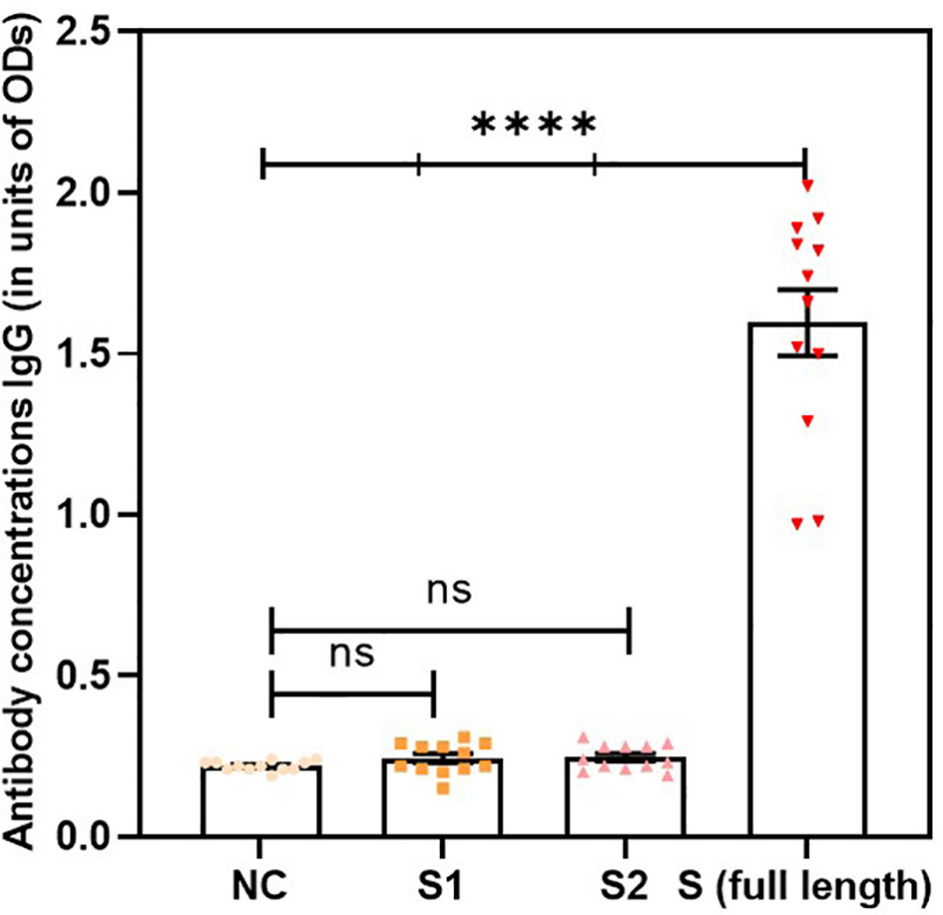
Significant antibody levels were detected for isotype class IgG in stimulated cells compared with the unstimulated negative control(NC) (p < 0.0001, n = 12). Data are expressed as the mean ± standard error of the mean (SEM). Comparisons between two groups were performed using paired *t-*tests.

**Fig. 2:**
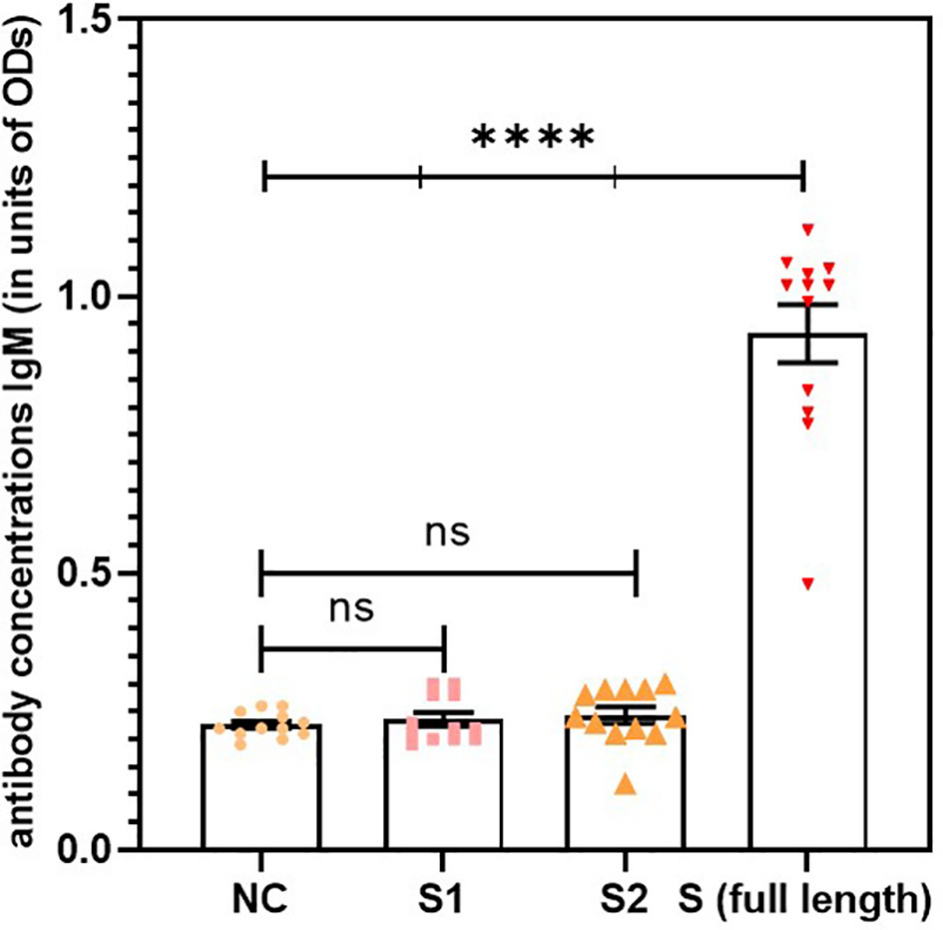
Significant antibody levels were detected for isotype class IgM in stimulated cells compared with the unstimulated negative control (NC) (p < 0.0001, n = 12). Data are expressed as the mean ± standard error of the mean (SEM). Comparisons between two groups were performed using paired *t-*tests.

**Fig. 3:**
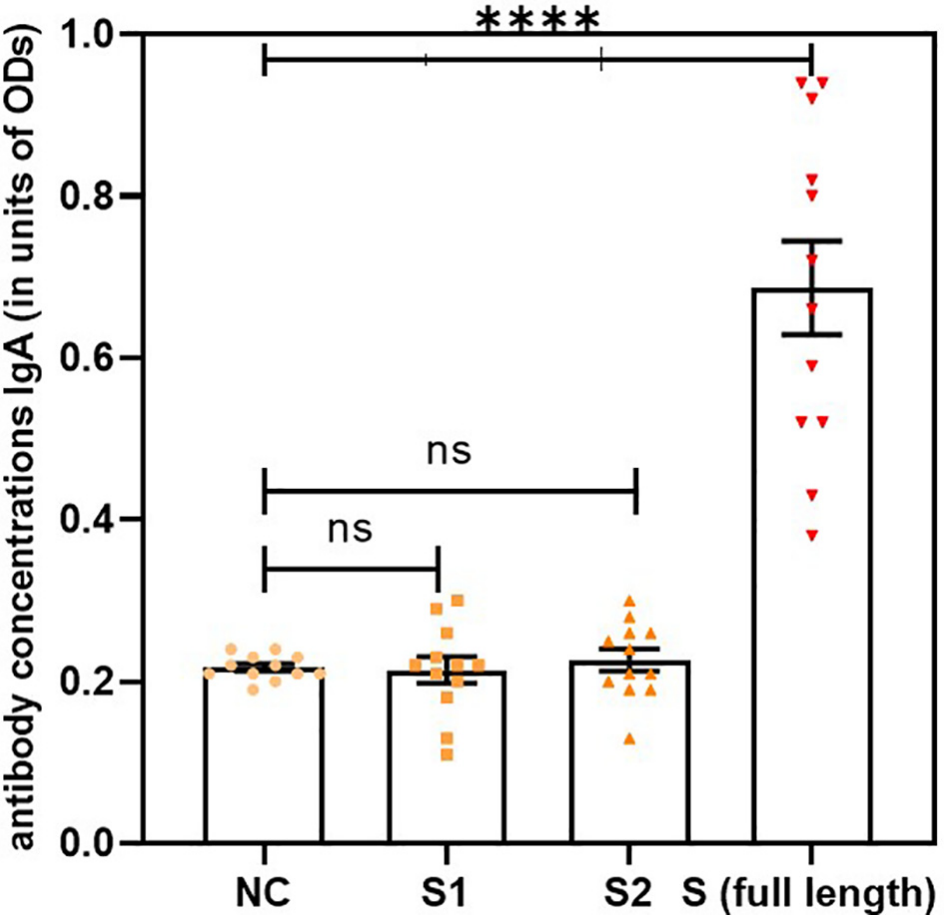
Significant antibody levels were detected for isotype class IgA in stimulated cells compared with the unstimulated negative control(NC) (p < 0.0001, n = 12). Data are expressed as the mean ± standard error of the mean (SEM). Comparisons between two groups were performed using paired *t-*tests.

SARS-CoV-2 S protein induced a strong humoral immune response in the NALT cell culture model. ELISA was used to measure the antibody levels in the cell culture supernatants at various time points. Significant antibody levels were detected at day 10 following the stimulation of MNCs with S protein compared with unstimulated negative control cells. Interestingly, all antibody classes were detected. Significant antibody levels were detected for isotype classes IgG, IgM, and IgA compared with those in the unstimulated negative control cells (Fig. 1, Fig. 2, Fig. 3, respectively, p < 0.0001, n = 12).

## 6 Discussion

The URT is the first interaction location for inhaled pathogens and intranasal vaccines. The URT contains a complex of lymphoid tissues, comprising draining lymph nodes and NALTs (Pizzolla et al., 2017). The lymphoid organs that have developed to service the URT enable the stimulation of immune responses to inhaled pathogens. We tested the involvement of these distinct lymphoid system components to study the humoral immune response following antigen stimulation. We found that NALTs served as a suitable human model for examining the immune response to respiratory pathogens, including the novel SARS-CoV-2. Improving the efficacy of intranasal vaccines depends on an improved understanding of the immune response, starting with antigen carriage into the URT.

Several well-established cell lines are commonly used to study respiratory viruses, derived from either animals, such as Vero and BGM from buffalo green monkeys (derived from kidney epithelium cells), or humans, including HeLa-T (derived from cervical epithelium cells) and Huh-7 (derived from liver hepatocellular carcinoma cells) (Kaye, 2006).

To the best of our knowledge, our study is the first study to use human NALT-derived MNCs as an *in vitro* human cell culture model to study the mucosal immune response to the SARS-CoV-2 S protein.

This novel model was used in a previous study and displayed a strong humoral immune response to live attenuated influenza virus (Mahallawi et al., 2013). The ability of this model to react and respond to antigens suggested that it could be used as a human model to study the natural immune response to various SARS-CoV-2 proteins, such as the S1 and S2 subunits and the full-length S protein.

Intranasal vaccination is more likely to resemble the natural infection process and has shown to be more potent than intravenous vaccination. Intranasal vaccination is a more biologically appropriate means of vaccination against respiratory infections, such as influenza and SARS-CoV-2 (Shim et al., 2020, Takaki et al., 2018). In terms of vaccine design, the normal infection route should be considered because it better mimics the process of natural infection. Our results revealed significant antibody production by NALT MNCs following stimulation with the SARS-CoV-2 S antigen.

NALT contains all of the immunocompetent cells that are essential for the generation of antigen-specific immune responses, which may be required for successful intranasal vaccination. Nasal vaccination has been confirmed to be an efficacious method for the stimulation of the respiratory immune system (Chin-ichi Tamura 2004). Furthermore, mucosal vaccination has been shown to provoke both humoral and cell-mediated antigen-specific immune responses (Yanagita et al., 1999) . As an additional benefit, nasal vaccination requires a smaller antigen quantity to stimulate antigen-specific mucosal and systemic immune responses than other vaccination routes (Gwinn et al., 2010).

Our result demonstrated the ability of the full-length SARS-CoV-2 S protein to induce a potent mucosal humoral immune response for IgG, IgM, and IgA antibody isotypes. Conversely, the S1and S2 subunit antigens failed to stimulate humoral immune cells to produce antibodies. The full-length spike protein comprises both subunits (S1 and S2) and is, therefore, larger in size (1209 amino acids) than either individual subunit (S1 contains 681 amino acids and S2 contains 539 amino acids). Moreover, the full-length S protein contains more epitopes than either the S1 or S2 individually. Neither subunit S1 nor S2 was sufficient to generate an immune response alone. The S1 and S2 antigens might be able to induce an immune response if combined with adjuvants, which are compounds that are added to vaccine antigens to ease and improve the activation of the innate and adaptive immune responses and can expand the immunogenicity and efficiency of vaccines (Reimer et al., 2012). Vaccines targeting the SARS-CoV-2 S protein are being developed and tested by several companies, such as Altimmune, CanSino biologicals, Moderna, and Novavax (Salvatori et al., 2020). The current intranasally delivered ChAd SARS-CoV-2 has been shown to be able to prevent both upper and lower respiratory tract infections and may potentially be protective against SARS-CoV-2 infections and transmission in mice (Hassan et al., 2020).

Our previous findings confirmed the suitability of using human NALT to study the interactions between influenza virus proteins and the immune system, demonstrating the applicability of this unique model to explore the humoral immune response to several influenza antigens (Mahallawi et al., 2013). In the current study, using the SARS-CoV-2 S protein, we demonstrated the potential for this novel model to be developed for the future study of cellular immune responses to spike and other viral proteins. The NALT model has previously been extensively studied using other viral and bacterial respiratory pathogens (Zhang et al., 2002, Zhang et al., 2011, Gray et al., 2014, Aljurayyan et al., 2018).

The ability of the S protein to stimulate the production of all three antibody isotypes by NALT-derived B-cells suggests that this system can be used for the in-depth investigation of the immune response to other SARS-CoV-2 antigens, including the M and N proteins. Several studies have demonstrated that a large fraction of B lymphocytes are present in the tonsils (Passali et al., 2003). Furthermore, CD4^+^ lymphocytes represent a primary component of tonsillar tissues. A low ratio of CD8^+^ cytotoxic lymphocytes was also found in these tissues (BERNSTEIN, 1999, BERNSTEIN et al., 1999).

We also found that the specific anti-S IgG antibody level was significantly higher than the other IgM and IgA isotypes, although both IgM and IgA levels in stimulated cells were significantly higher than those in unstimulated negative controls. A previous study reported that B-cells of the IgG isotype were predominant in tonsillar tissues, whereas B-cells of the IgM and IgA isotypes were comparatively less well represented (Edwards et al., 1986).

Our study was associated with several limitations. We did not investigate the cellular immune response using the current model. Additionally, we did not test other viral proteins, such as the N and M proteins. Last, we hypothesize that the use of adjuvants, such as CpG oligodeoxynucleotides, aluminum hydroxide, and others, might induce a stronger immune response, which may allow the S1 and S2 protein subunits to stimulate the production of antibodies by NALT MNCs, further increasing the S protein-induced humoral immune response; however, we did not test this theory in the current study.

## Declaration of Competing Interest

The authors declare that they have no known competing financial interests or personal relationships that could have appeared to influence the work reported in this paper.
